# Comparing analgesic efficacy of regional blocks after modified radical mastectomy: important issues should be noticed

**DOI:** 10.1186/s13741-024-00387-7

**Published:** 2024-05-17

**Authors:** Yu-Jing Yuan, Fu-Shan Xue, Tian Tian

**Affiliations:** 1 Department of Anesthesiology, Beijing Friendship Hospital, Capital Medical University, Beijing, 100050 China; 2 Department of Anesthesiology, Shengli Clinical Medical College of Fujian Medical University, Fujian Provincial Hospital, No. 134 Dongjie, Gulou District, Fuzhou, 350001 China

**Keywords:** Analgesia, Transverses thoracic muscle plane-pectoral nerve block, Thoracic paravertebral nerve block, Modified radical mastectomy, Early recovery

Dear Editor,

By conducting a randomized controlled trial with 80 female breast cancer patients who underwent the unilateral modified radical mastectomy, Zhao et al. (2022) compared the postoperative analgesic efficacy between the ultrasound-guided transverses thoracic muscle plane-pectoral nerve block (TTP-PECS) and thoracic paravertebral nerve block (TPVB) and showed that the TTP-PECS provided an improved postoperative analgesia, with a decreased opioid consumption and an increased the quality of recovery 40 (QoR-40) score. Other than the limitations described by the authors in the “Discussion” section, however, we noted several issues in the design and results of this study and wished to get the authors’ comments.

First, when a randomized controlled trial was designed to differentiate the effect of an intervention on the primary outcome, all of the other factors that influence the primary outcome assessment should be standardized for the avoidance of potential bias. The objects of this study were breast cancer patients undergoing the unilateral modified radical mastectomy. However, the authors did not provide the details of preoperative chemotherapy, radiotherapy and pain, and surgical procedures in the demographic and surgical characteristics of patients. Available evidence indicates that preoperative pain severity, use of analgesics or radiotherapy, size of tumors, and number of dissected lymph nodes are significantly associated with the occurrence of acute and chronic pain after breast cancer surgery (Habib et al. 2019; Klein et al. 2021; Schreiber et al. 2019). We are concerned that these unknown factors would have biased the main and secondary findings of this study.

Second, postoperative pain visual analog scale (VAS) scores at rest and during activity at 12 h postoperatively were significantly lower in the patients receiving the TTP-PECS compared with the patients receiving the TPVB. This indicates that the use of TTP-PECS tends to improve postoperative pain control. However, we noted that the net between-group differences in mean postoperative pain VAS scores at 12 h postoperatively were very small, with 0.49 at rest and 0.74 during activity. They are significantly less than the recommended minimal clinically important difference for acute postoperative pain control, i.e., 1.5 on a 0–10 VAS (Doleman et al. 2021). Furthermore, postoperative pain VAS scores at rest and during activity in most observed points postoperatively were only about 3 or less, indicating that most patients had a satisfactory postoperative pain control. Unlike the recent other works assessing postoperative analgesic efficacy of nerve blocks in patients undergoing modified radical mastectomy (Wu et al. 2022; Chai et al. 2022), this study did not assess patient satisfaction with postoperative pain control. In this case, it is difficult for the readers to determine whether early postoperative pain control improved by the TTP-PECS compared with the TPVB should be considered as being clinically important.

Third, the postoperative analgesia regimen of the two groups was an opioid-based intravenous analgesia, that is, patient-controlled analgesia using fentanyl, with mean fentanyl consumption of up to 547.3–701.0 μg within the first 24 h postoperatively. This does not meet the requirements of current enhanced recovery after surgery practices for breast cancer surgery, which recommend an opioid-sparing multimodal strategy containing a series of non-opioid analgesics with different mechanisms, such as intravenous/oral acetaminophen, ketorolac or ibuprofen, and gabapentin (Lee et al. 2022). Most importantly, non-opioid analgesics should be started to give before or during surgery and regularly repeated after surgery. Intravenous/oral opioids may be reserved for breakthrough pain only when non-opioid basic analgesics are ineffective or contraindicated. We believe that different results about comparison of postoperative analgesic efficacy between the TTP-PECS and TPVB would have been obtained, if a standard opioid-sparing multimodal analgesic strategy was included in this study design. Most importantly, this design limitation may have hindered the generalization of their findings into the current enhanced recovery after surgery practices.

Fourth, the total QoR-40 score was significantly higher in the patients receiving the TTP-PECS than in those receiving the TPVB, suggesting that the TTP-PECS provides an improved quality of early postoperative recovery. As a randomized controlled trial, however, the authors did not provide if the preoperative QoR-40 scores were comparable between groups. Furthermore, the net between-group difference in the means of total QoR-40 scores at 24 h postoperatively was 7.39, which does not exceed the recommended minimal clinically important difference of QoR-40 score for breast cancer surgery required in a randomized clinical trial, i.e., a 10-point difference (Altıparmak et al. 2020). In addition, the mean QoR-40 score at 24 h postoperatively in the patients receiving the TTP-PECS was 176.1, indicating that patients did not achieve a good quality of early postoperative recovery with a median QoR-40 score of 185 or more (Myles et al. 2016). Thus, we argue that the between-group difference in the quality of postoperative recovery is statistically significant, but its clinical significance is debatable.

Finally, other than the quality of early postoperative recovery, this study did not observe other outcome variables of current enhanced recovery after surgery practices for breast cancer surgery, such as the incidence of postoperative nausea and vomiting, the occurrence of postoperative complications, the time to early mobilization, and the time to hospital discharge (Lee et al. 2022). Because of this limitation, an important issue that this study cannot answer is whether improved pain control, decreased opioid consumption and enhanced early postoperative recovery by the TTP-PECS compared to the TPVB can be transformed into postoperative benefits of patients undergoing the unilateral modified radical mastectomy.

## Data Availability

Not applicable.

## Abbreviations

TTP-PECS: Transverses thoracic muscle plane-pectoral nerve block
TPVB: Thoracic paravertebral nerve block
VAS: Visual analog scale
QoR-40: Quality of recovery 40
